# Bimodal Porosity and Stability of a TiO_2_ Gig-Lox Sponge Infiltrated with Methyl-Ammonium Lead Iodide Perovskite

**DOI:** 10.3390/nano9091300

**Published:** 2019-09-11

**Authors:** Salvatore Sanzaro, Federico Zontone, David Grosso, Thomas Bottein, Fortunato Neri, Emanuele Smecca, Giovanni Mannino, Corrado Bongiorno, Corrado Spinella, Antonino La Magna, Alessandra Alberti

**Affiliations:** 1 National Research Council-Institute for Microelectronics and Microsystems (CNR-IMM), Zona Industriale-Strada VIII n°5, 95121 Catania, Italy or emanuele.smecca@imm.cnr.it (E.S.); giovanni.mannino@imm.cnr.it (G.M.); corrado.bongiorno@imm.cnr.it (C.B.); corrado.spinella@imm.cnr.it (C.S.); antonino.lamagna@imm.cnr.it (A.L.M.); 2 Department of Mathematical and Computational Sciences, Physics and Earth Sciences, University of Messina, Viale F. Stagno d’Alcontres 31, 98166 Messina, Italy; fneri@unime.it; 3 ESRF, The European Synchrotron, CS40220, 38043 Grenoble CEDEX 9, France; zontone@esrf.fr; 4 Institut Matériaux Microélectronique Nanosciences de Provence (IM2NP) Aix-Marseille Université, 13397 Marseille CEDEX 20, France; david.grosso@univ-amu.fr (D.G.); thomas.bottein@im2np.fr (T.B.)

**Keywords:** Ti-oxides, perovskites, blend, nano-scale, sputtering, Thornton, shadowing

## Abstract

We created a blend between a TiO_2_ sponge with bimodal porosity and a Methyl-Ammonium Lead Iodide (MAPbI_3_) perovskite. The interpenetration of the two materials is effective thanks to the peculiar sponge structure. During the early stages of the growth of the TiO_2_ sponge, the formation of 5–10 nm-large TiO_2_ auto-seeds is observed which set the micro-porosity (<5 nm) of the layer, maintained during further growth. In a second stage, the auto-seeds aggregate into hundreds-of-nm-large meso-structures by their mutual shadowing of the grazing Ti flux for local oxidation. This process generates meso-pores (10–100 nm) treading across the growing layer, as accessed by tomographic synchrotron radiation coherent X-ray imaging and environmental ellipsometric porosimetry. The distributions of pore size are extracted before (>47% V) and after MAPbI_3_ loading, and after blend ageing, unfolding a starting pore filling above 80% in volume. The degradation of the perovskite in the blend follows a standard path towards PbI_2_ accompanied by the concomitant release of volatile species, with an activation energy of 0.87 eV under humid air. The use of dry nitrogen as environmental condition has a positive impact in increasing this energy by ~0.1 eV that extends the half-life of the material to 7 months under continuous operation at 60 °C.

## 1. Introduction

Titanium dioxide (TiO_2_), since the discovery of its application to water photolysis by Fujishima and Honda in 1972 [1], has progressively raised interest in many different fields such as for photocatalytic degradation of pollutants [2,3,4], photocatalytic CO_2_ reduction into energy fuels [5,6,7,8], water splitting [9,10,11], solar cells and photonics [12,13], sensors [14,15,16], supercapacitors [17,18], biomedical devices [19,20,21] and lithium-ion batteries [22,23,24].

In the photovoltaic field, recent advances were linked to the application of porous TiO_2_ in hybrid solar cell architecture with efficiency that consequently went up strikingly rapidly [25] thanks to the new ideas of M. Grätzel (dye solar cells, DSC) [26,27] and T. Miyasaka (perovskite solar cells, PSC) [28,29,30]. DSCs, with their structure and operation mechanism [31,32,33,34,35,36,37,38], represent the precursor technology of PSCs that, in their original architecture, have a mesoporous TiO_2_ layer as scaffold to exchange the photo-generated carriers. Collection and injection efficiencies, comprising the parasitic diode operation, are indeed related to the structural properties of this scaffold. Additionally, since perovskite are required to form an interconnected blend with its scaffold, issues related to the accessibility of the TiO_2_ pores, effectiveness of the perovskite reaction [39,40], and the stability and durability of the blend [41,42,43,44,45,46,47,48,49,50,51,52,53] need to be faced. In this respect, the pore filling capability is a parameter of high technological impact [54].

The most diffused and versatile way to grow a mesoporous TiO_2_ scaffolds is by chemical methods. They can generate a large plethora of fascinating hierarchical and mesoporous structures [55,56,57] with high infiltration capability.

Standard physical growth methods are, instead, less performing in creating porous structures, and they are mostly based on the semi-empirical Thornton’s approach [58,59]. As a step forward, cauliflower [60], penniform [61] or zig-zag [62] structures can be found in the literature. Some other attempts to increase the TiO_2_ layer porosity were done using Ti nano-structures [14,63,64,65,66,67,68,69,70] deposited by sputtering in glancing angle configuration (GLAD) [71] and subsequently oxidized by ex-situ processes. Other approaches in the literature are based on the use of an array of template materials (e.g., polystyrene nano-spheres) to exploit their shadowing effect during TiO_2_ growth [72]. All the existing approaches add complexity to the fabrication process (need of external oxidation or templating layers or seeds), in a countertrend with the request of high production throughput.

In our previous work [48], we initiated a method called gig-lox (grazing incidence geometry with local oxidation) to grow multi-porosity oxide layers by modified sputtering processes and demonstrated that gig-lox-based PSCs exhibit comparable efficiency with respect to devices made with standard chemically synthetized TiO_2_ nano-structures, even if scaling down the thickness of the scaffolding layer. In addition, the electrical behavior of functionalized TiO_2_ gig-lox layers unveiled [49] its high injection capability and high collection efficiency of photo-generated carriers. The method used is up-scalable, reliable and indeed industrially implementable, solvent-free, and can be, in principle, extended to any reactive metallic source to produce porous oxides. The multi-scale-porosity (micro and meso) represents a key parameter to allow material of different size (and nature) intimately entering into the TiO_2_ scaffold and forming an active interacting (chemically and physically). In this respect, disclosing the early stage of formation of the gig-lox bimodal porosity and its capability to establish stable blends with photo-active perovskites would open the field for its industrialization in view of high throughput and indeed of low-cost production of solar devices.

In this framework, the paper illustrates the structural properties of the gig-lox TiO_2_ sponge since the early stage of the material growth. We illustrate the mechanism of growth by auto-seeds and we disclose the bimodal pore structure (micro- and meso-pores [73,74]) using advanced technique such as tomographic coherent synchrotron X-ray-based diffraction imaging (CXDI) and environmental ellipsometric porosimetry (EEP). The capability of the gig-lox structure to host hybrid perovskite materials (i.e., CH_3_NH_3_PbI_3_, hereafter called MAPbI_3_, being the most explored for light harvesting) is here exploited and investigated. The gig-lox process is conceived to be compatible with any kind of substrate and is up-scalable for high production throughput. We demonstrate that our gig-lox TiO_2_ scaffold blended with MAPbI_3_ has a high stability and durability, and that the degradation process is mostly related to the eventual presence of an adverse environment rather than to the multiple interfaces established inside the blend. The mainstays of the growth procedure could be extended to other oxides of technological interest.

## 2. Materials and Methods

### 2.1. TiO_2_ Gig-Lox Deposition

TiO_2_ gig-lox layer is deposited in a customized DC-pulsed (Direct Current) sputtering equipment (Kenosistec S.r.l., Milano, Italy) we exploited the grazing incidence geometry of the titanium source assisted by the local oxidation (gig-lox) of the Ti species landing on the substrate to grow porous TiO_2_ layers with multiple size porosity [73,74]. The deposition method and its mainstays are fully described in our previous papers [37,48,49].

### 2.2. Perovskite Preparation

Perovskite materials were deposited by solution processing on TiO_2_ gig-lox layer, deposited by DC-pulsed sputtering on corning glass substrates. After TiO_2_ deposition, a thermal annealing at 500 °C for 5 min in simulated dry air (80% N_2_ and 20% O_2_) was performed on the sample to assure anatase phase formation. Before perovskite deposition, the sample was treated by UV-O_3_ for 10 min to clean the surface and avoid the presence of contaminants. Deposition of the perovskite films was performed in a dry room with a monitored humidity (~20%) by a Cl-assisted one step deposition. A 40 wt% solution of PbCl_2_ and methyl-ammonium iodide (MAI) (ratio 1:3) in dimethyl-formamide (DMF) was prepared at 70 °C under stirring for 1 h (Sigma Aldrich Co., St. Louis, MO, USA). The solution was spin coated on TiO_2_ at 1200 rpm for 30 s; after that the rotational speed was increased to 2000 rpm for 6 s and subsequently to 4500 rpm for 24 s. During spin coating, 1 mL of toluene was dripped onto the substrate as solvent treatment to form a homogenous film. The substrates were then placed on a hot plate which was raised up to 90 °C for 40 min, up to 100 °C for 10 min and finally to 110 °C for 10 min to complete the evaporation of the solvent [42]. The as-deposited MAPbI_3_ layers have comparable average thickness (~350 nm). The sample was stored in vials under a dry nitrogen atmosphere immediately after preparation to avoid triggering any degradation before the analyses.

### 2.3. X-ray Reflectivity and X-ray Diffraction Method

X-ray reflectivity (XRR) and X-ray diffraction (XRD) measurements were done using a D8-Discover Bruker AXS diffractometer equipped with a Cu-K_α_ source/Goebel mirror (Bruker Corporation, Billerica, MA, USA) and eventually soller slits at the primary beam, and with variable slits and detector at the secondary path.

### 2.4. Transmission Electron Microscopy Method

Transmission electron microscopy (TEM) analyses were done in plane-view and cross-section using a JEOL JEM 2010 microscope operating at 200 kV (JEOL Ltd., Akishima, Tokyo, Japan).

### 2.5. Scanning Transmission Electron Microscopy Method

Scanning transmission electron microscopy (STEM) images were acquired at 200 kV in scanning mode using a high angle annular dark field detector in Z-contrast configuration using a Jeol ARM200 equipped with a cold FEG electron source, CEOS condenser aberration corrector and at 100 mm^2^ (JEOL Ltd., Akishima, Tokyo, Japan).

### 2.6. CXDI Method

CXDI analysis was performed using the equipment located at the beamline ID10 at the European Synchrotron Radiation Facilities in Grenoble, France (ESRF, Grenoble, France). The strength of the CXDI technique resides in the ability of high-resolution imaging, in principle limited by the highest q-vector where speckles are measurable. CXDI relies on a numerical algorithm that allows to phase the Fourier space to the real space when speckles are over-sampled [75,76,77]. CXDI is a powerful tool for providing a 3D image of the outer and the inner structures of micro-particles with a resolution of a few tens of nanometers [75,78] thus giving access to the full description of the morphology of the particles like porosity and specific surface area.

Fragments of TiO_2_ scaffolds were dispersed on the surface on a Si_3_N_4_ membrane via a gentle mechanical scratching of the support. Isolated micrometric particles were selected using an on-axis optical microscope. The measurements were performed using 8.1 keV radiation produced by the undulator source of the ID10, monochromatized by a Si(111) pseudo channel-cut monochromator and focused by compound refractive lenses at the sample location. The final beam was defined by a 10 μm square-shaped aperture of rollerblade slits and had an average intensity of 6 × 10^10^ ph/s. Tomographic series of 2D coherent small-angle speckle patterns were recorded by a Maxipix detector with 516 × 516 pixels located 3.280 m downstream the sample, resulting in a voxel size of 17.8 nm in the real space reconstructions and a maximum field-of-view of ~3 μm (linear oversampling ~3). The tomographic scans around the normal to the membrane surface were spanning an angular range of 162° for the particles of TiO_2_ and TiO_2_ with PbI_2_, 144° for the particle with MAPbI_3_, all of them with 0.25° angular step. A beam-stop prevented the direct beam to damage the detector with only a partial obstruction of the central speckles thanks to a careful alignment. The 2D projections were assembled to build the 3D Fourier space with 512 × 512 × 512 resolution elements that was phased by a combination of hybrid input–output and error-reduction algorithms [78]. Finally, 20 reconstructions were averaged to account for high frequency variations in the convergence due to noise. The conversion into absolute units was performed according to the beamline parameters (incidence intensity and beam size) and exposure time of the single frames (27 s for the TiO_2_ particle, 15 s for the other two, TiO_2_ with MAPbI_3_ and TiO_2_ with PbI_2_). 

### 2.7. Spectroscopic Ellipsometry Method

Spectroscopic ellipsometry (SE) data were collected using a J.A. Woollam VASE instrument (J.A. Woollam Co., Lincoln, NE, USA). Measurements were performed in a vertical configuration, which is better suited for transparent samples in order to measure on the same point ellipsometric and transmittance data. Optical spectra were recorded from 300 to 2100 nm (step 5 nm) at 55°, 60° and 65°. An initial model of the optical transitions was built for each layer constituting the sample. The TiO_2_ layer was modelled by using a single Tauc-Lorentz oscillator and the surface roughness by the Bruggeman effective medium approximation (EMA). The technique was mainly used to measure the average porosity of the layer.

### 2.8. EEP Method

EEP was investigated using a visible (from 370 to 1000 nm) variable angle spectroscopic ellipsometer (ESM 300 Woollam) with an atmospheric control chamber [79] (J.A. Woollam Co., Lincoln, NE, USA). Isopropanol (Sigma Aldrich Co., St. Louis, MO, USA) was selected as the adsorbate and was fluxed into the chamber over the film. The volume of adsorbed and capillary condensed isopropanol was followed through time-resolved measurement of the refractive index variation upon the relative pressure of the adsorbate (P/P_0_). The volume of adsorbate was deduced from the evolution of the refractive index using the Cauchy models and the Bruggeman Effective Medium Approximation (J.A. Woollam CompleteEASE software). The pore size distribution was then plotted using Kelvin’s equation and by using a model of cylindrical pores.

## 3. Results and Discussion

### 3.1. Auto-Seeds Formation: XRR and TEM Analyses

**Figure 1:**
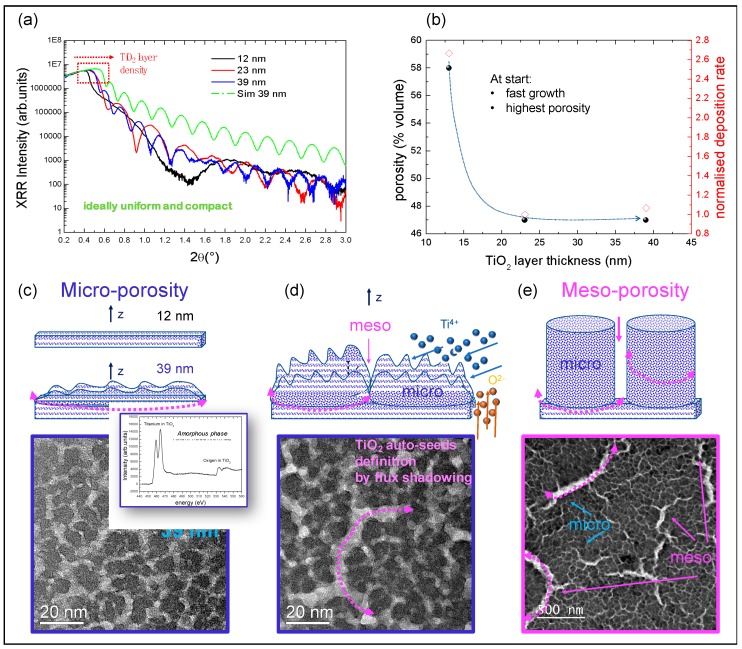
(**a**) X-ray reflectivity (XRR) profile of starting layers of different thickness, compared to the ideal case of a compact flat amorphous TiO_2_ layer in the anatase polymorph. (**b**) porosity and deposition rate as a function of the grown thickness. Plane-view transmission electron microscopy (TEM) analyses of (**c**) a layer containing auto-seeds (39 nm); (**d**) a thicker layer with meso-pores starting to be defined; (**e**) a final layer (400 nm) with meso- and micro-porosity defined on a large scale (cross-section in Figure 2). The schematic provides the overall scenario during the initial growth, starting from the first minutes (transient) wherein a matrix of small TiO_2_ grains and micro-pores are equally distributed and originated in a Thornton-like regime (high Ar pressure). During further growth, a network of larger TiO_2_ auto-seeds gives rise to grazing incidence geometry with local oxidation (gig-lox) columns separated by meso-pores due to shadowing under rotation (steady state growth).

We started by investigating the early stages of the material growth to disentangle the assembly process. We stopped the growth at the first 12 nm, 23 nm and 39 nm of material, as measured by SE and XRR. The XRR curves acquired on those samples are shown in Figure 1a in comparison with the simulated profile of an ideal flat compact TiO_2_ layer supposed to have anatase density [15]. In the figure, it is worthy to note that the critical angle for total external reflection, linked to the square root of the electronic surface density of the material, in the thinnest layer is largely under the reference (simulated profile in Figure 1a) while it progressively increases during further growth without ever matching the ideal value. This finding implies that the electronic density of the material is less than what is exposed in a compact amorphous TiO_2_ phase, conversely providing indication that a porous layer has been grown. According to this, the experimental XRR curves can be shaped using electronic density and roughness as main fitting parameters. The average porosity values, in agreement with the SE findings, are shown in Figure 1b using the equation:

(1) $$P\left(\%V\right)=\left[1-\left(\frac{\rho_{\mathrm{sample}}}{\rho_{\mathrm{bulk}}}\right)\right]\times 100,$$

where in ρ_sample_ is the measured density and ρ_bulk_ is the density of the compact TiO_2_ reference.

In the same graph, the deposition rate, normalized to the final plateau value (4 nm/min), was superimposed using red symbols. The cross-correlation of the data provides the scenario of a TiO_2_ growth occurring through: (1) a first (transient) regime wherein the deposition rate is high and this corresponds to the maximum porosity value; (2) a second regime in which porosity and deposition rate approach steady-state values of ~47% volume and ~4 nm/min, respectively. The presence of a first layer with high porosity (~12 nm-thick) was included for fitting the profiles of thicker layers. Above this porous basement, nanometric auto-seeds (10–20 nm in diameter, separated by gaps, namely micro-pores, initially of 2–10 nm in size) are statistically generated introducing a surface roughening in the layer (Figure 1c). Their growth, according to the gig-lox method [37], is assisted by a progressive bottom-up local oxidation (lox) via an independent oxygen flux impinging onto the growing front [48,49]. The sample is under rotation during growth to assure a uniformity in thickness with a proper internal nano-structuration. The establishment of tens-of-nm-large TiO_2_ auto-seeds on the sample surface is demonstrated by TEM analyses in plane-view, as that shown in Figure 1c done on the 39 nm-thick layer. In the inset, the electron energy loss spectroscopy (EELS) spectrum testifies a Ti:O = 1:2 stoichiometry and a mostly amorphous arrangement of the building-block. A macro-assembly process occurs during further growth by the progressive statistical drawing of meso-borders (meso-pores) by a shadowing action that selects the sites for ad-atoms attaching and finally brings together adjacent nano-seeds into larger meso-columns (Figure 1d,e). The resulting material is a TiO_2_ sponge with a double-scale porosity. Shadowing and local oxidation are the mainstays of the process that can be applied to any substrate and oxide for thickness up-scalable to (at least) 1000 nm. The final structure of TiO_2_ layers grown under steady-state conditions was described in refs [15,48,49] by TEM and SEM analyses and exploited for application in different fields. In what follows, insights on the double-scale porosity (meso and micro) will be gained by CXDI, a new methodology for imaging and tomography based on coherent synchrotron radiation, and by ellipsometric nano-porosimetry to explore to what extent the sponge can interconnect a perovskite material.

### 3.2. Bimodal Porosity Investigation

#### 3.2.1. CXDI Analyses

We imaged the 3D architecture of a TiO_2_ gig-lox layer 400 nm-thick, grown at the steady state, using non-conventional tomographic coherent X-ray diffraction imaging (3D-CXDI) based on synchrotron radiation [75,80]. CXDI works in ambient conditions and with an easier sample preparation that does not require specific treatments like tinning. It is a lens-less imaging technique where the electron density distribution of an isolated object in real space is retrieved by an iterative algorithm that phases an oversampled speckle pattern recorded in reciprocal space in the far field [75,76,77]. The technique provides the three-dimensional distribution of the electronic density on the basis of the local structure of the material (i.e., a combination of morphology and stoichiometry) with a resolution of a few tens of nanometers [78,81]. Here the electronic densities are given in absolute units (e^−^/Å^3^) after normalization of the diffraction patterns to the incident intensity and beam size (see Materials and Methods). The voxel (volumetric picture element, i.e., the pixel in the 3D space) size is 17.8 nm.

**Figure 2:**
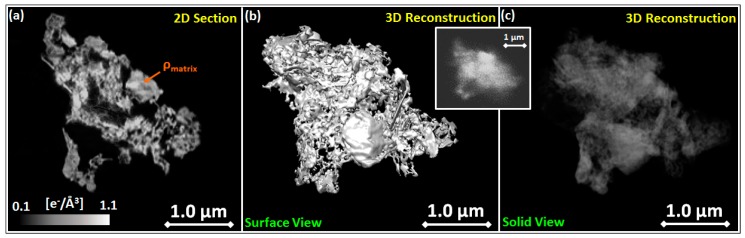
Coherent X-ray diffraction imaging (CXDI) reconstruction of a TiO_2_ gig-lox portion representing: (**a**) central section showing the density ρ_matrix_ being smaller than anatase; (**b**) 3D reconstruction showing the morphology of the sample at its surface (surface view) and (**c**) 3D reconstruction based on the integrated electron density (see also Figure 5). Inset: Environmental Scanning Electron Microscopy (ESEM) image used as benchmark of the CXDI reconstruction.

3D-CXDI has been applied to image isolated fragments of TiO_2_ layers deposited on a Si_3_N_4_ membrane (see Video S1 as Supplementary Material). Before the measurements, the sample was annealed at 500 °C in order to promote a lattice order in the anatase polymorphism without significantly upsetting the layer porosity [15,38,48]. Fragments of dimension in the range 2–5 μm were selected by optical inspection to meet the oversampling requirements (see Experimental Methods). Figure 2 shows a section (slice), the 3D image and the 3D electronic density distribution of a fragment of a pure TiO_2_ scaffold. The reconstruction reveals a highly heterogeneous structure with pores having a large distribution in size, inherent to the gig-lox deposition method. With the voxel size of 17.8 nm, 3D-CXDI is well suited to image the meso-porosity both by slicing the fragment or by the 3D-reconstruction.

Additionally, we evaluated the average meso-porosity of the sponge within the fragments by computing the surface-to-volume ratio (SVR) of the reconstructed images using the Chimera [82] software (Figure 2b). The obtained value of 0.06 nm^−1^ corresponds to the SVR expected from a rods structure with rods having radius ~33 nm [48] according to the expression for a cylindrical shape:

(2) $$\frac{S}{V}=\frac{2\pi rL}{\pi r^{2}L}=\frac{2}{r}=0.06\;{\mathrm{nm}}^{-1}\to r=33\;\mathrm{nm}.$$

The extracted radius accounts for the meso-structure of the material as described by the TEM cross-section images [48]. 

Micro-porosity cannot be directly resolved by 3D-CXDI; nevertheless, it has an indirect impact on the absolute value of the electronic density, being consequently lower than the anatase reference. A lower density of the matrix relates to the degree of porosity (see Figure 2a), according to Equation (1), modified as follows [83]:

(3) $$P\left(\%V\right)=\left[1-\left(\frac{\rho_{\mathrm{matrix}}}{\rho_{\mathrm{anatase}}}\right)\right]\times 100,$$

where ρ_matrix_ and ρ_anatase_ are the locally sampled electronic densities of the porous TiO_2_ and anatase (1.08 e^−^/Å^3^), respectively. The matrix component is extracted by the “Trainable Weka Segmentation” plugin in the Fiji software [84]. By creating a histogram of the matrix voxel values over all the reconstruction slices, the average electronic density can be determined. We found an average electronic density of the TiO_2_ scaffold ρ_matrix_ = 0.63 e^−^/Å^3^, indicating a degree of porosity (P(%)), P (%) ~42%, a value which is in agreement with the data in Figure 1a after auto-seeds formation, witnessing the sensitivity of 3D-CXDI to micro-porosity.

To disclose and support the relationship between micro- and meso-porosity [73,74], we complemented the CXDI analysis with EEP and scanning-TEM investigations.

#### 3.2.2. EEP Analyses

EEP is used to quantitatively measure the layer porosity at the nano-scale and to gain insight on the pore size distribution, especially for the small pores. This technique measures the changes of the refractive index during adsorption and capillary condensation of a solvent (here isopropanol) into the pores of the material. The results are shown in Figure 2. The volume fraction of adsorbed isopropanol is deduced from the Bruggeman effective medium approximation (BEMA) which compares the refractive index of the empty film (void + TiO_2_) with that of the film full of isopropanol (isopropanol + TiO_2_) [79]. In this calculation, the fraction of TiO_2_ remains fixed and the fraction of voids (accessible porosity) is considered to be equal to the fraction of isopropanol. The analysis was performed using a refractive index of 2.65 for the as-deposited sample, supposing a mixture of anatase and rutile in the local ordering of the amorphous phase; for the samples annealed at 500 °C sample, being purely anatase [37], we used a refractive index of 2.53.

**Figure 3:**
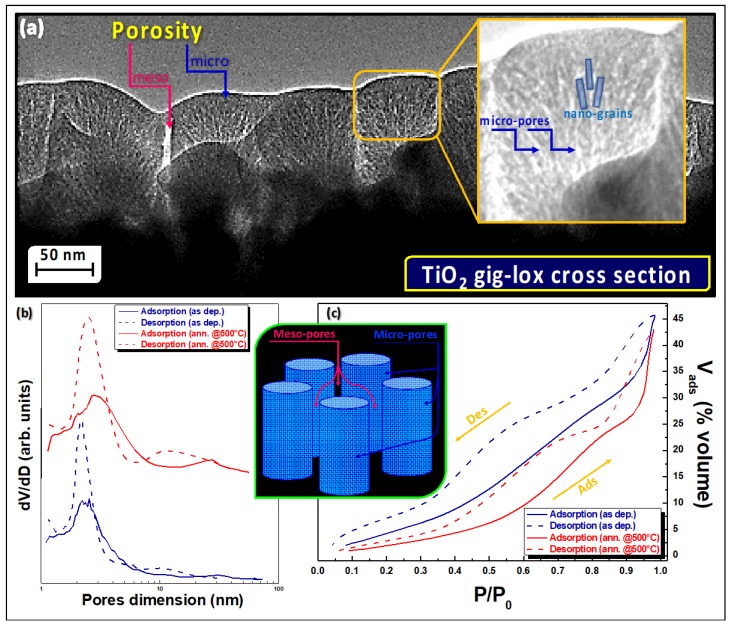
(**a**) TEM cross section of the gig-lox layer showing the material arrangement by nano-grains in a matrix of interconnected micro-pores (zoom in the yellow region). (**b**) Pore size distribution deduced from the Kelvin law for cylindrical interconnected pores for TiO_2_ gig-lox in as-deposited and after annealing at 500 °C conditions. (**c**) Volume of adsorbed gas as a function of P/P_0_ for TiO_2_ gig-lox in as-deposited and after annealing at 500 °C conditions.

Based on what was previously found [48], cylindrical pores will be used for the calculation of pore size distribution (pore aspect ratio = 10). To a direct comparison, the TEM cross-section image in Figure 3a is additionally provided with details meso-/micro-pores [73,74] and on the internal nano-structuration of the TiO_2_ columns (see Figure 1). Pore size distribution (in terms of their diameter) is given in adsorption and in desorption conditions. In adsorption, the average pore diameter is probed while in desorption the average diameter of the interconnection between pores is determined. In Figure 3b that the plot dV/dD gives the distribution of pore size diameter as a function of the number of pores [79]. Large pores will be less visible on this graph even if they can represent a large volume of the total porosity. Indeed, for a given contribution in term of volume, the number of large pores will be lower than the number of small pores. 

The pore size distribution in Figure 3b highlights some main points: (1) the bimodal distribution of the pores; (2) the persistence of small-size pores even after the thermal treatment at 500 °C; (3) a slight enlargement of the distribution after annealing and a small shift of the maximum rightwards that accounts for a slight increase of the average pore size (further discussed in the next paragraphs). Those small changes are mainly related to the thermal contraction of TiO_2_ occurring during crystallization to anatase (a further contribution of pore sintering is not excluded).

In order to get a good vision over the pore distribution in term of volumetric percentage, it is preferable to consider the plot of Vads as a function of P/P_0_ (where P_0_ is the absolute pressure of the adsorbate) [79]. The total percentage of porosity slightly decrease after annealing at 500 °C. The bimodal distribution of the pores is visible by the changes in the slope around a partial pressure of 0.7 (P/P_0_ = 0.7) corresponding to small pores and around a partial pressure of 0.9 (P/P_0_ = 0.9) corresponding to large pipelines. Roughly half of the porosity (in volume) is accounted for by the large pores and half by the small pores. By comparison between the refractive index of the layers before and after isopropanol adsorption and the bulk refractive index of the material, we deduce that a part of the porosity is occluded (not probed by isopropanol). In particular, 20% of the empty volume is not accessible before annealing. After annealing, the fraction of occluded porosity diminishes to less than 10% of the material volume likely due to a contraction of the TiO_2_ matrix with temperature.

Furthermore, those results demonstrate that the hierarchical porous network is well interconnected as smaller and larger pores (main large pipelines) are probed via diffusion and condensation of isopropanol. For further structural confirmation, see the inset in Figure 3a. 

### 3.3. MAPbI_3_ Loading

**Figure 4:**
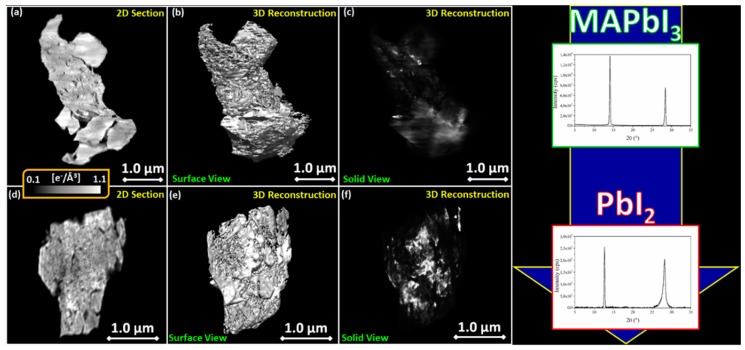
CXDI images of (**a**–**c**) a blend of TiO_2_ and MAPbI_3_; (**d**–**f**) a blend of TiO_2_ and PbI_2_. The brilliant zones in (**c**,**f**) correspond to material with high electronic density (>anatase, TiO_2_). As representative of the others, in(**f**) the brilliant rows (regions of density above 0.9–1 e^−^/Å^3^) of PbI_2_ embedded into the matrix mark the infiltration path of MAPbI_3_ into the vertical meso-pipelines. The color bar refers to all 2D sections.

MAPbI_3_ was deposited to enter the gig-lox structure via solution processing, as described in the methods section. The upper inset in Figure 4 shows the diffraction pattern of the synthesized perovskite with its typical tetragonal structure [44]. Fragments of the MAPbI_3_/TiO_2_ blend were prepared and analyzed by 3D-CXDI similarly as done for pure TiO_2_ (see Figure 2). The advantage of using this approach for analyses resides in the lack of any special preparation procedure, as instead needed for TEM analyses; and this is extremely crucial when dealing with hybrid perovskites such as MAPbI_3_ to avoid immediate degradation of the sample [85]. Representative reconstructions are shown in Figure 4. The upper raw refers to a MAPbI_3_/TiO_2_ blend after deposition. The sections (see Figure 4a) locally reveal a largely compact morphology with an average volumetric electron density substantially higher than that of the empty TiO_2_ scaffold. The compactness of the sample (see Figure 4b) and the raised density account for the perovskite having filled the mesopores of the scaffold during the solution processing. To see how MAPbI_3_ entered the porous TiO_2_ scaffold, we applied the Weka segmentation method to isolate the matrix and quantify the average electron density. This was 0.88 e^−^/Å^3^, 40% larger than what found for the TiO_2_ particles. The expected density for a pure MAPbI_3_ sample is 1.05 e^−^/Å^3^, while the TiO_2_ scaffold has ρ_matrix_ = 0.63 e^−^/Å^3^. Figure 4c also reveals clusters at high electronic density (>1.3 e^−^/Å^3^), likely accounting for a local sporadic compositional change in the perovskite layer towards PbI_2_ [42,44,45,48] (the electronic density of PbI_2_ is 1.51 e^−^/Å^3^).

The lower row of Figure 4e,f shows the effect of the full degradation of MAPbI_3_ into PbI_2_ (yellow sample) after prolonged exposure of the sample to air (see also the related XRD pattern in the inset). For this sample, the electronic density distribution of Figure 4f depicts a scenario of PbI_2_ distributed all over the sample volume, with part of the iodide percolated inside vertical mesopores of the TiO_2_ scaffold.

**Figure 5:**
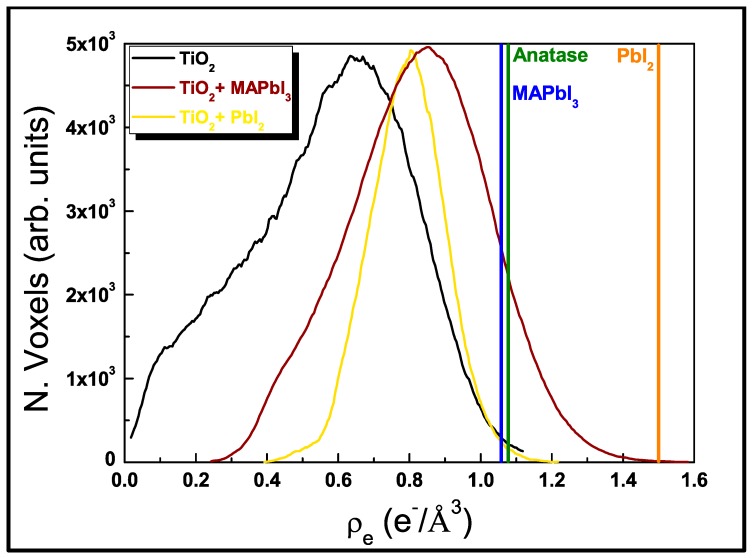
Electronic density distribution measured by CXDI in pure gig-lox TiO_2_, in the TiO_2_–MAPbI_3_ blend and in the TiO_2_–PbI_2_ blend.

Figure 5 plots the histograms of the matrix of the three investigated samples: the pure gig-lox scaffold; the scaffold infiltrated with perovskite (starting blend) and the blend after degradation. Through the distribution, a rationalization on the perovskite infiltration capability is drawn. The data additionally depict a scenario on how the perovskite modifies inside the TiO_2_ scaffold as an effect of ageing in air. On this basis, the electronic density in the pure TiO_2_ scaffold has a large distribution with mean value at 0.63 e^−^/Å^3^ and width (FWHM) ~0.4 e^−^/Å^3^, which can be viewed as a combination of micro-porosity (the mean value) and meso-porosity (the width) [73,74]. The distribution intercepts the compact anatase density at the high-density tail. After blending with the perovskite, the histogram shifts rightwards since the loading increases the average electron density. At this stage, the percentage of free volume drops from P ~42% to P ~18%. We point out that this last value could be overestimated with respect to the starting blend condition since even a small degradation of the perovskite in the fragment due to ambient exposure [44,45] would notably increase the fraction of the empty volume (a volume contraction by 54% occurs as a MAPbI_3_ degrades to PbI_2_). As soon as the phase conversion to PbI_2_ is completed by prolonged exposure (several days), the histogram moves back leftwards. The minor extent of this shift depends on the interplay between the higher electronic density of the PbI_2_ and the increase in porosity arising from the volume contraction during degradation, producing an average electron density slightly smaller (0.80 e^−^/Å^3^). The Perovksite back-transition to PbI_2_ can be easily identified inside the fragment in the 3D-reconstruction of CXDI data (solid view), and this especially traces the starting distribution of the host material through the mesopores (vertical brilliant zones in Figure 4f).

**Figure 6:**
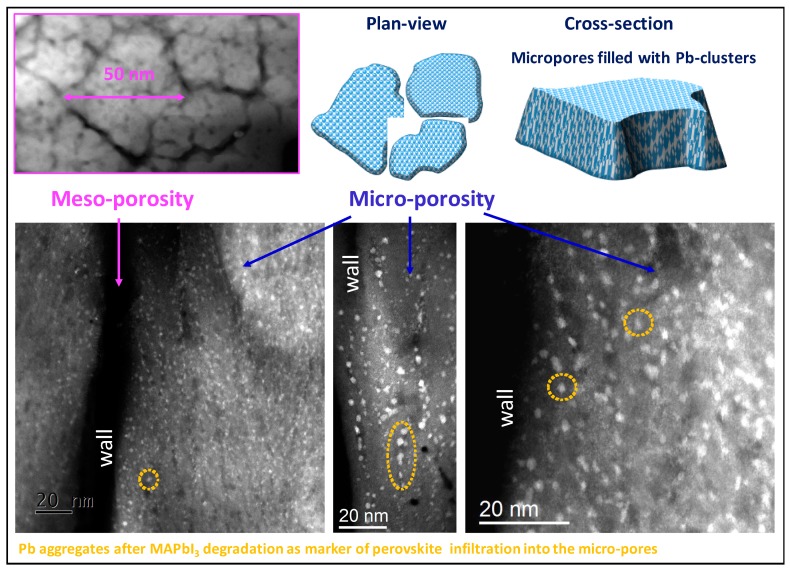
Scanning transmission electron microscopy images of the TiO_2_–MAPbI_3_ blend after degradation of the perovskite to PbI_2_ and Pb aggregation. Pb nanoclusters are used as markers to trace the initial distribution of the perovskite into the micro-pores (some of them are circled in yellow).

Resolving the perovskite distribution inside the micro-pores is beyond the resolution of the CXDI measurements. Therefore, scanning TEM (STEM) analyses were used to complement the information at the nano-scale. Following the approach adopted in the CXDI-based diagnostic for meso-pores, a marker was used to follow the infiltration path in the nano-gaps. A sample was, at this purpose, prepared by focused ion beam thinning procedure and analyzed by STEM in cross-section at a base pressure of ~10^−9^ Torr. Under these adverse conditions the perovskite layer has a reduced lifetime [86] and even Pb nanoclusters can be formed [46]. We exploited the MAPbI_3_ conversion into Pb-nanoclusters to trace the pristine perovskite distribution into the sponge. The method is especially effective to highlight micro-pores since the size of the Pb-clusters is comparable to the pore size. Deep and capillary perovskite soaking was indeed testified by the spreading of those Pb-nanoclusters through the whole layer along rows that are made brilliant in STEM by the high mass of the lead atoms. Some representative images are shown in Figure 6. 

### 3.4. MAPbI_3_/TiO_2_ Blend Stability

**Figure 7:**
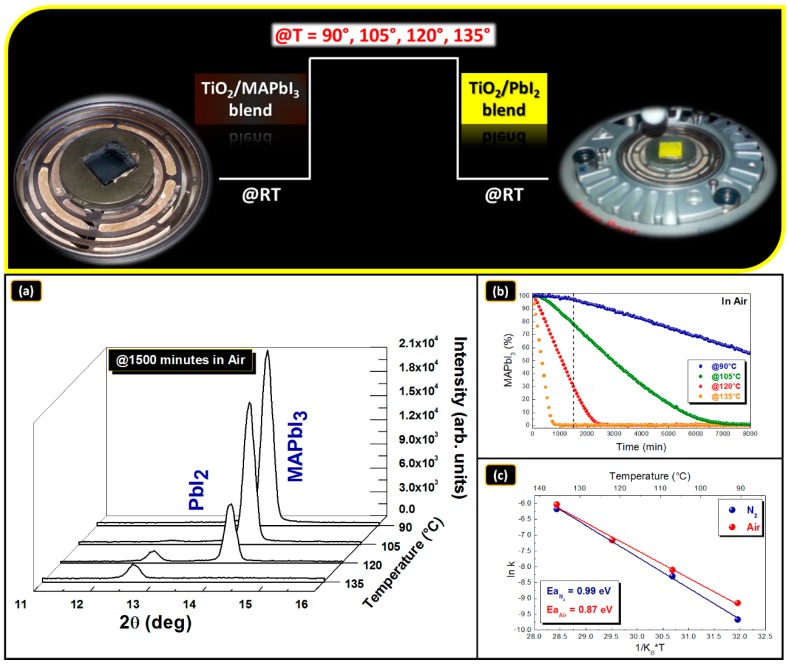
(**a**) XRD patterns collected after at 1500 min annealing in air at different temperatures; (**b**) kinetic analysis and fitting of the degradation curves of blend samples annealed at four different temperatures (90 °C, 105 °C, 120 °C and 135 °C) in air; (**c**) Arrhenius plot of the kinetic constant and activation energy extracted in air and dry N_2_ environment. All data were collected in dark conditions.

One main concern on the blend to be applied is its durability under sun irradiation. This is firstly because the temperature of the blend is expected to increase with a possible impact on its stability. The maximum temperature during operation under the sun is expected to be in the range of 60–80 °C. In the experiment designed to test the thermal stability of the MAPbI_3_–TiO_2_ blend, the sample was kept under isothermal annealing in the range of 90–135 °C in controlled atmosphere, as schematically depicted in Figure 7. The range was chosen to apply a stress test to mimic prolonged operation conditions. As a testing procedure, the structural modification of the perovskite was traced by collecting its diffraction pattern during time at a fixed temperature, while the diffraction chamber was kept at 55 ± 5% HR (relative humidity), intentionally under the threshold to form hydrated perovskites [87,88]. A fresh sample was used for each temperature. During annealing and in a time frame proportional to the temperature used, all infiltrated materials were seen to progressively change their composition from MAPbI_3_ to PbI_2_. Consistently, the color of the sample changed from brown to yellow as shown in the upper panel of Figure 7. This was testified by the progressive consumption of the MAPbI_3_ area of the main peak at 2θ = 14.00° ((002)/(110)) and the simultaneous increase of the PbI_2_ peak at 2θ = 12.6° ((003) planes of the 9R hexagonal structure).

We measured the ((110)/(002)) MAPbI_3_ peak area during time (Figure 7a) and converted this value into a mass variation according to [89] and applying the equation:

(4) $$y=\left[{\mathrm{MAPbI}}_{3}\;\%\right]=\left[\left(\frac{A_{0}}{A_{i}\left(t\right)}\right)\times 100\right],$$

where A_0_ is the area of the (002) peak at t = 0 and Ai is the corresponding area at t = t_i_. The parameter can be used as a marker for the material degradation on the basis of some experimental observations, i.e., excluding a re-orientation of the original crystal lattice and with texturing maintained as in the starting condition. With this method, we find out that the degradation curves follow a decreasing trend. In Figure 7b we show the data recorded in air conditions, namely in the presence of moisture. For all the temperatures analyzed, the rate of the degradation is constant and thus the curves follow zero order kinetics. The kinetic constant *k* governing the process was extracted as:

(5) $$y=\left[{\mathrm{MAPbI}}_{3}\;\%\right]=\left[k\left(T\right)\times t\right].$$

Used in an Arrhenius plot as

(6) $$k\left(T\right)=k_{0}e^{-\frac{E_{a}}{K_{B}T}},$$

where K_B_ is the Boltzmann constant, the parameter can be applied to extract the activation energy of the degradation process. The value obtained for degradation reaction of the MAPbI_3_ in air ambient is ~0.87 eV. This energy can be associated to a mechanism of degradation via volatilization of species from the lattice, being in the range of typical dissociation energies reported in the literature [41]. Although the value for perovskite degradation in the blend is lower by ~0.1 eV than that found in a flat layered material, the degradation times are roughly comparable (due to the non-negligible role of the pre-exponential factor) as commented in what follows. In the degradation process, MAI and/or HI, being both volatile species, are expected as by-products [90] mostly in the presence of water molecules, that have a catalytic action in activating their formation [42,91]. As a matter of fact, the identical experiment done in dry nitrogen is found to be governed, temperature by temperature, by lower kinetics constants with an associated activation energy of 0.99 eV.

In their summary, our findings not only shine light on the degradation mechanism of the perovskite in the blend, which is similar to what occurs in layers of perovskites, but additionally have a predictive role on what is expected in the operation range of temperatures under adverse (humid air) vs. inert (N_2_) conditions. It must indeed be noticed that the two curves in Figure 7c tend to diverge at low temperatures, and this accounts for a better stability of the material under nitrogen conditions. We summarized the half-life times of the perovskite in Table 1:

The table highlights the stabilization action of the overall blend via dry nitrogen and this suggests that the multiplication of interfaces with TiO_2_, expected as instability sources, plays a minor role with respect to the environment. This demonstrates that our blend made of the gig-lox TiO_2_ scaffold plus the infiltrated perovskite has a durability useful for application. Moreover, the half-life time measured at a working temperature of 60 °C under air conditions is comparable to that exhibited by a similar perovskite layer deposited on flat TiO_2_ as reported in our previous work [42]. This indeed implies that a blended architecture based on the gig-lox scaffold can be alternatively applied to standard layered architecture, with advantages likely offered by the extended surfaces available for charge extraction and collection. 

## 4. Conclusions

To conclude, in exploring the early stages of the growth of the gig-lox TiO_2_ sponge, auto-seeds are found to frame the bimodality of the generated pores. At first, a transient condition is established wherein the deposition rate is high and this corresponds to the maximum measured porosity. After that, moving towards a steady-state condition, hundred-nm auto-seeds are statistically generated and selected for further growth by the inclined ad-atoms and the shadowing effect. The resulting meso-grains contain the original nano-seeds and micro-porosity defined during the early stages of growth. In both steps, a bottom-up oxidation process is assured by a piloted oxygen path, independent from the Ar path and flow.

At the steady-state of the growth process, an overall porosity above 47% is established in the layer. Tomographic X-ray imaging (CXDI) acquired with synchrotron radiation on fragments of samples revealed a meso-porosity in the range ~10–100 nm that was subsequently exploited for perovskite loading. CXDI 3D-Imaging depicted a scenario of large perovskite infiltration along the meso-pores by mapping the electronic density distribution per unit volume into fragments of the blend, with pore filling estimated above 80% in volume. Although micro-pores are under the resolution of the technique, their accessibility is testified through complementary STEM analyses. MAPbI_3_ conversion to PbI_2_ or Pb was detected by CXDI and STEM with spatial distribution and resolution at the meso and micro scales, respectively, for the rationalization on the high capability of a gig-lox TiO_2_ scaffold to form an intercalated blend with state-of-the-art hybrid photo-active materials.

The degradation of the perovskite in the blend, explored by a stress test experiment, follows a degradation path towards the formation of PbI_2_ as solid by-product with the concomitant release of volatile species that need an activation energy sensitive to the environment used. The presence of humid air and nitrogen differently impacts on the value, with a delta of ~0.1 eV in favor of the inert environment that extends the half-life of the material to 7 months under continuous operation at 60 °C. The degradation process is indeed triggered by the environmental composition (e.g., humid air) rather than being related to the multiple interfaces established inside the blend.

## Figures and Tables

**Table 1:** Half-life time of the perovskite in the blend under continuous operation at the indicated temperatures.

T (°C)	N_2_	Air
30	8854 days	1410 days
60	210 days	70 days
80	25 days	12 days

## Supplementary Materials

The following are available online at https://www.mdpi.com/2079-4991/9/9/1300/s1, Video S1: TiO_2_ 3D-Reconstruction by CXDI.
